# Evaluation of α-tubulin, detyrosinated α-tubulin, and vimentin in CTCs: identification of the interaction between CTCs and blood cells through cytoskeletal elements

**DOI:** 10.1186/s13058-018-0993-z

**Published:** 2018-07-05

**Authors:** G. Kallergi, D. Aggouraki, N. Zacharopoulou, C. Stournaras, V. Georgoulias, S. S. Martin

**Affiliations:** 1 Laboratory of Τumor Cell Biology, School of Medicine, University of Crete, Heraklion, Greece; 2 Department of Biochemistry, University of Crete, Greece Medical School, Heraklion, Greece; 3 Department of Physiology, Marlene and Stewart Greenebaum Comprehensive Cancer Center, University of Maryland School of Medicine, 655 W. Baltimore Street, Baltimore, MD USA

**Keywords:** CTCs, Microtentacles, α-Tubulin, Detyrosinated α-tubulin, Vimentin, Breast cancer, Cytoskeleton, Metastasis

## Abstract

**Background:**

Circulating tumor cells (CTCs) are the major players in the metastatic process. A potential mechanism of cell migration and invasion is the formation of microtentacles in tumor cells. These structures are supported by α-tubulin (TUB), detyrosinated α-tubulin (GLU), and vimentin (VIM). In the current study, we evaluated the expression of those cytoskeletal proteins in CTCs.

**Methods:**

Forty patients with breast cancer (BC) (16 early and 24 metastatic) were enrolled in the study. CTCs were isolated using the ISET platform and stained with the following combinations of antibodies: pancytokeratin (CK)/VIM/TUB and CK/VIM/GLU. Samples were analyzed with the ARIOL platform and confocal laser scanning microscopy.

**Results:**

Fluorescence quantification revealed that the ratios CK/TUB, CK/VIM, and CK/GLU were statistically increased in MCF7 compared with more aggressive cell lines (SKBR3 and MDA-MB-231). In addition, all of these ratios were statistically increased in MCF7 cells compared with metastatic BC patients’ CTCs (*p* = 0.0001, *p* = 0.0001, and *p* = 0.003, respectively). Interestingly, intercellular connections among CTCs and between CTCs and blood cells through cytoskeleton bridges were revealed, whereas microtentacles were increased in patients with CTC clusters. These intercellular connections were supported by TUB, VIM, and GLU. Quantification of the examined molecules revealed that the median intensity of TUB, GLU, and VIM was significantly increased in patients with metastatic BC compared with those with early disease (TUB, 62.27 vs 11.5, *p =* 0.0001; GLU, 6.99 vs 5.29, *p =* 0.029; and VIM, 8.24 vs 5.38, *p =* 0.0001, respectively).

**Conclusions:**

CTCs from patients with BC aggregate to each other and to blood cells through cytoskeletal protrusions, supported by VIM, TUB, and GLU. Quantification of these molecules could potentially identify CTCs related to more aggressive disease.

**Electronic supplementary material:**

The online version of this article (10.1186/s13058-018-0993-z) contains supplementary material, which is available to authorized users.

## Background

Μetastasis, rather than the primary tumor, is mainly responsible for cancer-related death. The metastatic process is associated with the presence of circulating tumor cells (CTCs) and disseminated tumor cells in peripheral blood and bone marrow, respectively [1, 2]. CTCs hold stem and epithelial-to-mesenchymal transition (EMT) properties, which are difficult to target with common chemotherapeutic agents [3–5]. The malignant nature of CTCs is supported by the presence of chromosomal alterations and by xenograft mouse models [6–9]. However, some of them are dormant or apoptotic [10, 11], and it seems that only a small proportion of CTCs are capable of forming overt tumor deposits [12].

CTCs are an extremely heterogeneous population; therefore, it is crucial to isolate and effectively characterize CTCs according to their tumorigenic capacity [12]. We have reported that CTCs express growth factor receptors and activated signaling kinases such as epidermal growth factor receptor, human epidermal growth factor receptor 2 (HER2), phosphorylated phosphatidylinositol 3-kinase, p-AKT, and p-FAK [13, 14]. However, it has been shown that there are important phenotypic and biological discrepancies between CTCs and patients’ primary tumors, implying that it is crucial to characterize these cells and use them as potential targets for cancer treatment [13, 15–17]. To this end, we have reported that it is possible to improve patients’ outcomes by targeting CTCs rather than primary tumors and prevent tumor cell spreading [18].

A mechanism for metastatic dissemination is the formation of microtentacles. These cytoskeletal structures are supported by α-tubulin (TUB) and associated with the EMT pathways [19, 20]. Vimentin (VIM), Twist, and Snail are particularly upregulated in microtentacle-expressing cells. Furthermore, cancer cells with the capacity for cell migration and invasion are characterized by stem cell phenotype and microtentacle protrusions [19–24]. Detyrosinated α-tubulin (GLU) is another interesting characteristic of these cytoskeletal structures, considering the fact that GLU is a poor prognostic factor for patients with positive primary tumors [25].

Recent evidence indicates that common chemotherapeutic agents such as taxanes cause shedding of CTCs into the bloodstream, which can dramatically increase cancer spread and relapse [26, 27]. Taxanes can also increase microtentacles, promoting tumor cell reattachment [28]. However, other drugs such as kinesin inhibitors or curcumin can diminish microtentacles and inhibit tumor cell dissemination [22, 29].

The characterization of the microtentacles’ structural proteins in isolated CTCs from patients with breast cancer (BC) has not been extensively addressed so far. The goal of the current study was to identify these molecules on isolated CTCs and to explore their potential interference with the metastatic process. Finally, we investigated possible implications of microtentacles in inter-CTC communication and in CTC-to-blood cell crosstalk.

## Methods

### Cell cultures

Three different BC cell lines, representative of distinct subtypes, were used to create an expression pattern of the assessed molecules: MCF7 (hormone receptor-positive [HR^+^]), SKBR3 (HER2^+^), and MDA-MB-231 (basal-like). All cell lines were obtained from the American Type Culture Collection (Manassas, VA, USA). The MCF7 cells were cultured in 1:1 (vol/vol) DMEM/Ham’s F-12 medium (Life Technologies, Carlsbad, CA, USA) supplemented with 10% FBS (Life Technologies), 2 mM L-glutamine (Life Technologies) 30 mM NaHCOB_3B_, 16 ng/ml insulin, and 50 mg/ml penicillin/streptomycin (Life Technologies). SKBR3 cells were cultured in McCoy’s medium (Life Technologies) enriched with 10% FBS and 2 mM L-glutamine supplemented with 50 mg/ml penicillin/streptomycin. MDA-MB-231 cells were cultured in high-glucose DMEM (Life Technologies) with 10% FBS and 2 mM L-glutamine supplemented with 50 mg/ml penicillin/streptomycin. Cells were maintained in a humidified atmosphere of 5% CO_2_/95% air. Subcultivation for all cell lines was performed with 0.25% trypsin and 5 mM ethylenediaminetetraacetic acid (EDTA). All experiments were performed during the logarithmic growth phase. For spiking experiments, various dilutions (10 cells/ml, 100 cells/ml, and 1000 cells/ml of blood) of cells from three cell lines were spiked in 10 ml of blood obtained from healthy blood volunteers.

### Patients’ blood samples

Peripheral blood (10 ml in EDTA) was obtained from 16 chemotherapy-naïve patients with early BC and 24 patients with metastatic disease, before the initiation of any line of treatment, according to the design of a previous study [30]. Patients without evidence of metastatic disease (stages I–II) were considered to have early BC, whereas patients with stage IV disease were included in the metastatic group. Most of the patients were postmenopausal (62.5% early and 58.3% metastatic) in both cohorts. The HR^+^ type comprised 75% of adjuvant and 62.5% of metastatic subjects. Triple-negative tumors (HR^−^HER2^−^) were represented in 6.3% of the patients with early disease and in 25% with metastatic disease. Sixteen patients from the metastatic group were initially diagnosed with early and operable disease, and seven other patients were diagnosed with metastatic disease from the beginning of the study. All the patients’ characteristics are shown in Table 1.

Blood samples were collected at the middle of vein puncture after the first 5 ml of blood were discarded in order to avoid contamination of the blood sample with epithelial cells from the skin during sample collection. This protocol was approved by the ethics and scientific committees of our institution, and all patients and healthy blood donors gave their informed consent to participate in the study.

**Table 1:** Patients’ characteristics

Early disease (16 patients)		Metastatic disease (24 patients)	
Age, yr, median (range)	53 (33–77)	Age, yr, median (range)	58 (39–70)
	No. (%)		No. (%)
Menopausal status		Menopausal status	
Premenopausal	4 (25%)	Premenopausal	7 (29.2%)
Postmenopausal	10 (62.5%)	Postmenopausal	14 (58.3%)
Unknown	2 (12.5%)	Unknown	3 (12.5%)
Tumor size		Tumor size	
pT1	9 (56.3%)	pT1	4 (16.7%)
pT2	5 (31.3%)	pT2	12 (50%)
pT3	0 (0%)	pT3	4 (16.7%)
Unknown	2 (12.5%)	Unknown	4 (16.7%)
Lymph node status		Lymph node status	
Node-negative	4 (25%)	Node-negative	9 (37.5%)
Node-positive	11 (68.8%)	Node-positive	10 (41.7%)
Unknown	1 (6.3%)	Unknown	5 (20.8%)
Histologic grade		Histologic grade	
Grade 1	0 (0%)	Grade 1	0 (0%)
Grade 2	10 (62.5%)	Grade 2	12 (50%)
Grade 3	3 (18.8%)	Grade 3	9 (37.5%)
Grade 4		Grade 4	3 (12.5%)
Unknown	3 (18.8%)	Unknown	0 (0%)
Histologic subtype		Histologic subtype	
Ductal	12 (75%)	Ductal	17 (70.8)
Lobular	1 (6.3%)	Lobular	2 (8.3%)
Other	3 (18.8%)	Other	5 (20.8%)
ER/PR tumor status		ER/PR tumor status	
Positive	12 (75%)	Positive	15 (62.5%)
Negative	2 (12.5%)	Negative	6 (25%)
Unknown	2 (12.5%)	Unknown	3 (12.5%)
HER2 tumor status		HER2 tumor status	
Positive^a^	7 (43.8%)	Positive^a^	3 (12.5%)
Negative	6 (37.5%)	Negative	18 (75%)
Unknown	3 (18.8%)	Unknown	3 (12.5%)
HR^+^/HER2^−^	5 (31.3%)	HR^+^/HER2^−^	12 (50%)
HR^+^/HER2^+^	6 (37.5%)	HR^+^/HER2^+^	3 (12.5%)
HR^−^/HER2^+^	1 (6.3%)	HR^−^/HER2+	0 (0%)
HR^−^/HER2^−^	1 (6.3%)	HR^−^/HER2^−^	6 (25%)
Unknown combination	3 (18.8%)	Unknown combination	3 (12.5%)
		Disease sites	
		1	8 (33.3%)
		2	11 (45.8%)
		≥ 3	4 (16.6%)
		Unknown	1 (4.2%)
		Predominantly visceral disease	
		Yes	15 (62.5%)
		No	7 (29.1%)
		Unknown	2 (8.3%)
		Primary breast cancer	
		Adjuvant	16 (66.7%)
		Metastatic	7 (29.2%)
		Unknown	1 (4.2%)
		Line of treatment	
		First	8 (33.3%)
		Second	8 (33.3%)
		Third	3 (12.5%)
		Fourth or later	5 (20.8%)

*Abbreviations: ER* Estrogen receptor, *PR* Progesterone receptor, *HR* Hormone receptor, *HER2* Human epidermal growth factor receptor 2

^a^positive were considered all the patients with HER2 score +3 in immunohistochemistry staining or +2 with positive FISH

### ISET system isolation of circulating tumor cells

CTCs were isolated using the ISET (Isolation by SizE of Tumor cells) platform (Rarecells Diagnostics, Paris, France) according to the manufacturer’s instructions. This isolation system was chosen because in a previous study it was shown that the ISET platform has a high recovery rate of tumor cells, regardless of the BC subtype [31]. Briefly, 10 ml of peripheral blood were diluted in 1:10 ISET buffer (Rarecells Diagnostics) for 10 min at room temperature (RT), and 100 ml of the diluted sample was filtered using a depression tab adjusted at −10 kPa. The membrane was dried for 2 h at RT and stored at −20 °C. Each membrane spot was used for identification of CTCs after immunostaining and fluorescence microscopy analysis.

### Confocal laser scanning and Ariol system microscopy

The presence of CTCs on ISET spots was evaluated using A45-B/B3 mouse antibody (Micromet, Munich, Germany) detecting CK8, CK18, and CK19, along with CD45 antibody (common leukocyte antigen), in order to exclude possible ectopic expression of cytokeratins by hematopoietic cells. A patient was considered as CTC-positive only if she harvested CK^+^/CD45^−^ cells (Fig. 2d). In addition, the cytomorphological criteria followed by Meng et al. were also used in order to characterize a cell as CTCs [9].

Consequently, patients were analyzed for the expression of TUB, GLU, and VIM. Triple-staining experiments were performed with the following combinations of antibodies: CK/TUB/VIM and CK/GLU/VIM. The samples were subsequently evaluated using the Ariol system (Leica Biosystems, Buffalo Grove, IL, USA) and confocal laser scanning microscopy.

For CK/TUB/VIM immunofluorescence staining, spots were incubated with PBS for 5 min, and then cells were permeabilized with 2% Triton X-100 for 10 min. After 1 h blocking with PBS/10% FBS, cells were incubated with VIM antirabbit antibody (Santa Cruz Biotechnology, Santa Cruz, CA, USA), followed by Alexa Fluor 633 antirabbit secondary antibody (Life Technologies). Subsequently, samples were stained with TUB antimouse antibody (Sigma-Aldrich, Taufkirchen, Germany) and Alexa Fluor 555 antimouse secondary antibody (Life Technologies) for 45 min Zenon technology (fluorescein isothiocyanate-conjugated immunoglobulin G1 [IgG1] antibody; Molecular Probes, Eugene, OR, USA) was used for CK detection with the A45-B/B3 antibody. Zenon antibodies were prepared within 30 min before use [16].

For triple-staining of CK/GLU/VIM, the same blocking and permeabilization procedures were followed, and the membranes were incubated with A45-B/B3 mouse antibody for 1 h. Consequently, after incubation for 45 min with the secondary antibody (Alexa Fluor 488 antimouse; Life Technologies), cells were stained with GLU antirabbit antibody (Abcam, Cambridge, MA, USA) overnight. Subsequently, cells were incubated with Alexa Fluor 633 antirabbit antibody. Finally, Zenon technology (Alexa Fluor 555-conjugated IgG1 antibody) was used for VIM staining (Santa Cruz Biotechnology). Positive controls were also included in each experiment, using the aforementioned cell lines spiked in healthy volunteers’ blood, whereas negative controls were prepared by omitting the corresponding primary antibodies and incubating the cells with the matching IgG isotype bound to the corresponding fluorochrome. Each patient with at least one CTC belonging to a distinct phenotype was considered as positive for this phenotype.

### Statistical analysis

The criteria for the evaluation of objective response rate (ORR) were according to RECIST 1.1 (Response Evaluation Criteria In Solid Tumors): tumor size, lymph node status, lesion number, and so forth [32]. Overall survival (OS) was defined as the time from entrance into the study until death from any cause. Progression-free survival (PFS) was defined as extending from study enrollment until disease relapse or death, whichever occurred first. Kaplan-Meier curves and Cox regression analysis for PFS and OS were compared using the log-rank test to provide a univariate assessment of the prognostic value of selected clinical risk factors. Variables that were found to be significant in univariate analysis were then entered in a stepwise multivariate Cox proportional hazards regression model to identify those with independent prognostic value. All statistical tests were performed at the 5% level of significance. IBM SPSS Statistics version 22 software (IBM, Armonk, NY, USA) was used for the analysis.

## Results

### Evaluation of TUB, GLU, and VIM in BC cell lines

The expression of TUB, GLU, and VIM in MCF7, SKBR3, and MDA-MB-231 cell lines was initially assessed with spiking experiments followed by ISET system isolation. Triple-staining experiments revealed that the ratios CK/TUB, CK/GLU, and CK/VIM were significantly increased in the well-differentiated HR^+^ MCF7 cells compared with the more aggressive cell lines, such as SKBR3 and MDA-MB-231 (Table 2).

**Table 2:** Expression of α-tubulin, detyrosinated α-tubulin, and vimentin in cell lines and circulating tumor cells

	Ratio CK/TUB	Tubulin	Ratio CK/GLU	GLU	Ratio CK/VIM	Vimentin
MCF7	5.46 ± 0.7	30.8 ± 3.5	20.41 ± 0.6	9.08 ± 0.3	42.22 ± 2.8	4.27 ± 0.5
SKBR3	1.49 ± 0.7	29.37 ± 6.6	12.49 ± 0.9	12.89 ± 0.7	31.34 ± 2.4	4.94 ± 0.4
MDA-MB 231	3.09 ± 0.4	26.19 ± 3.1	11.225 ± 0.7	8.045 ± 0.2	3.46 ± 0.8	28.87 ± 3.7
CTCs in patients with early breast cancer	4.58 ± 0.4	11.50 ± 0.4	15.5 ± 0.6	5.29 ± 0.6	14.33 ± 0.6	5.38 ± 0.3
CTCs in patients with metastatic breast cancer	1.75 ± 0.4	62.27 ± 18.7	15.28 ± 2.8	6.99 ± 0.4	8.05 ± 1.9	8.24 ± 1
*t* tests	*p* values					
MCF7 vs MD-MB231	0.002	0.307	0.0001	0.0001	0.002	0.001
MCF7 vs SKBR3	0.0001	0.463	0.0001	0.005	0.011	0.235
MCF7 vs CTCs early	0.038	0.0001	0.185	0.0001	0.002	0.022
MCF7 vs CTCs metastatic	0.0001	0.0001	0.0001	0.0001	0.003	0.000
MDA-MB231 vs SKBR3	0.032	0.344	0.446	0.001	0.0001	0.0001
MDA-MB231 vs CTCs early	0.332	0.0001	0.001	0.0001	0.0001	0.0001
MDA-MB231 vs CTCs metastatic	0.0001	0.0001	0.000	0.124	0.000	0.0001
SKBR3 vs CTCs early	0.036	0.010	0.001	0.0001	0.001	0.429
SKBR3 vs CTCs metastatic	0.282	0.0001	0.002	0.0001	0.0001	0.0001
CTCs early vs CTCs metastatic	0.0001	0.0001	0.937	0.029	0.007	0.0001

*Abbreviations: CK* Cytokeratin, *TUB* α-Tubulin, *GLU* Detyrosinated α-tubulin, *VIM* Vimentin, *CTCs* Circulating tumor cells

TUB intensity did not differ significantly among the cell lines. The highest GLU expression was observed in SKBR3 cells, where it was significantly different from MCF7 and MDA-MB-231 cells. VIM was extremely high in MDA-MB-231 cells compared with MCF7 and SKBR3 cells.

Microtentacles were observed mainly in MDA-MB-231 cells. We also noticed that coincubation of cancer cells with blood samples resulted in the appearance of cytoskeletal bridges between cancer and blood cells. This communication was observed mainly in SKBR3 and MDA-MB-231 spiked samples (Additional file 1: Figure S1).

### Evaluation of TUB and CK/TUB ratio in CTCs isolated from patients with early and metastatic BC

**Fig. 1:**
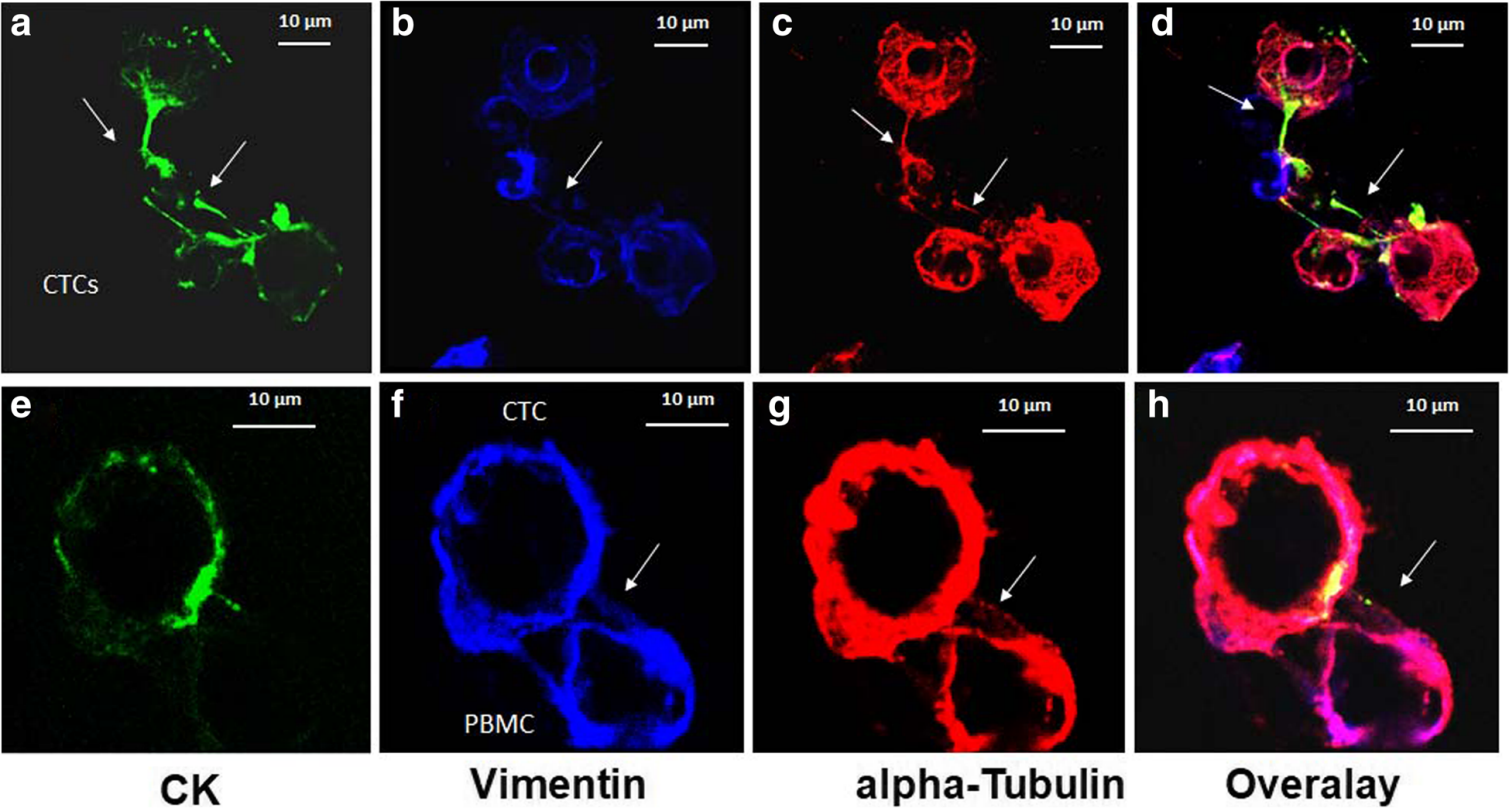
Expression of cytokeratin (CK), vimentin (VIM), and α-tubulin (TUB) in patients’ CTCs. Patients’ samples were stained with pancytokeratin (A45-B/B3) (green), vimentin (blue), α-tubulin antibodies (red), and 4′,6-diamidino-2-phenylindole (DAPI) (not shown). **a**–**d** Representative confocal laser scanning micrographs of patients’ CTCs (× 40). *White arrow* indicates the cytoskeleton bridges between CTCs supported by TUB, VIM, and CK. **e**–**h** Intercellular connections (*white arrows*) between a patient CTC and a blood cell (× 60). CTCs were positively stained for CK (green), TUB (red), and VIM (blue), whereas blood cells are positive for VIM and TUB

CTCs were detected in 11 of 16 (68.8%) and 16 of 24 (66.7%) patients with early and metastatic BC, respectively. The mean and median numbers of CTCs per patient were 4.6 and 1 (range, 0–37), respectively, for early BC, whereas in metastatic subjects, the corresponding numbers were 59.5 and 1.5 (range, 0–1062). Triple-staining experiments (TUB/VIM/CK) and confocal laser scanning analysis revealed that CTCs contacted each other through cytoskeletal bridges (Fig. 1a–d, white arrows). In addition, they communicated with microtentacle connections with nearby blood cells. These inter-CTC bridges supported by TUB, VIM, and cytokeratin (Fig. 1a–d). However, microtentacles that connected CTCs to blood cells (Fig. 1e–h, white arrows) were mostly supported by TUB and VIM. Each patient with at least one TUB^+^VIM^+^CK^+^ cell is considered as positive for this phenotype.

**Fig. 2:**
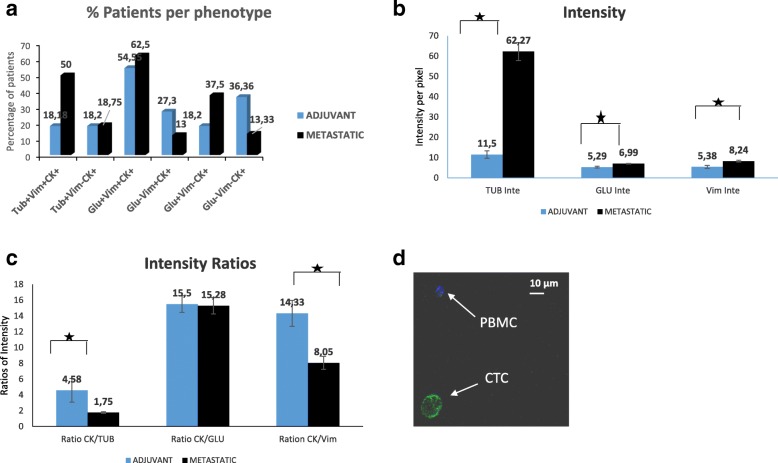
Quantification of cytokeratin (CK), α-tubulin (TUB), detyrosinated α-tubulin (GLU), and vimentin (VIM) in patients with early and metastatic breast cancer. **a** Percentage of the corresponding circulating tumor cell (CTC) phenotypes in patients’ blood. Each patient was considered as positive for a distinct phenotype if she harvested at least on CTC in her blood with this phenotype. **b** Quantification of TUB, GLU, and VIM intensity in CTCs derived from patients with early and metastatic breast cancer. **c** Quantification of CK/TUB, CK/GLU, and CK/VIM ratios in CTCs derived from patients with early and metastatic breast cancer. **d** Patient CTCs stained with pancytokeratin (A45-B/B3, green) antibody and CD45 (hematopoietic cell marker, blue) antibody

Ariol system analysis revealed that the phenotype (TUB^+^VIM^+^CK^+^) prevailed in CTCs from patients with metastatic disease (8 of 16 CK^+^ patients; 50%) compared with patients with early disease (2 of 11 CK^+^ patients; 18.2%) (*p =* 0.058) (Fig. 2a). Conversely, the incidence of the TUB^+^VIM^−^CK^+^ phenotype was not changed between the two groups (18.75% and 18.2%, respectively). The absolute number of CTCs per patient for each distinct phenotype is shown in Table 3.

**Table 3:** Number of circulating tumor cells per phenotype in each patient

Patients	TUB^+^VIM^+^CK^+^	TUB^+^VIM^−^CK^+^	GLU^+^VIM^+^CK^+^	GLU^−^VIM^+^CK^+^	GLU^+^VIM^−^CK^+^	GLU^−^VIM^−^CK^+^
Patients with early breast cancer						
1	0	13	8	0	16	0
2	0	0	0	0	0	0
3	0	0	0	0	0	0
4	1	0	12	0	4	0
5	1	0	1	0	0	0
6	0	0	0	2	0	0
7	0	0	1	0	0	3
8	0	1	0	0	0	1
9	0	0	0	1	0	0
10	0	0	0	0	0	1
11	0	0	0	0	0	0
12	0	0	0	0	0	0
13	0	0	0	0	0	1
14	0	0	1	0	0	0
15	0	0	0	0	0	0
16	0	0	1	5	0	0
Patients with metastatic breast cancer						
1	531		531	0	0	0
2	3	2	2	9	0	0
3	193	0	35	0	19	0
4	0	5	0	0	0	8
5	0	0	0	0	0	0
6	0	3	7	0	3	0
7	0	0	0	0	0	0
8	0	0	0	0	0	0
9	0	0	0	0	0	0
10	0	0	0	0	1	0
11	1	0	0	0	0	0
12	0	0	6	0	0	0
13	1	0	2	0	0	0
14	7	0	7	0	0	
15	0	0	0	0	0	0
16	0	0	0	0	0	0
17	16	0	7	0	7	
18	0	0	0	0	1	0
19	0	0	0	0	0	0
20	0	0	0	1	0	0
21	0	0	0	0	0	0
22	0	0	3	0	0	0
23	0	0	13	0	2	0
24	1	0	0	0	0	1

*Abbreviations: CK* Cytokeratin, *TUB* α-Tubulin, *GLU* Detyrosinated α-tubulin, *VIM* Vimentin

Quantification of TUB expression revealed statistically increased intensity in CTCs derived from patients with metastatic BC compared with all the examined cell lines (Table 2). Moreover, the intensity of TUB was statistically (*p =* 0.0001) lower in CTCs from patients with early BC (11.5 ± 0.4) compared with that observed in patients with metastatic disease (62.27 ± 18.7) (Table 2, Fig. 2b). Furthermore, the ratio CK/TUB was statistically lower (*p =* 0.0001) in metastatic patients’ samples (1.75 ± 0.4) compared with the MCF7 (5.46 ± 0.7) and MDA-MB-231 (3.09 ± 0.4) cell lines (Table 2).

The ratio of CK/TUB was significantly higher in CTCs detected in patients with early BC compared with that observed in CTCs from patients with metastatic disease (4.58 ± 0.4 vs 1.75 ± 0.4; *p =* 0.0001) (Table 2; Fig. 2c).

The distribution of all the CTCs regarding TUB intensity and CK/TUB ratio in both groups compared with MCF7, SKBR3, and MDA-MB-231 is shown in Additional files 2 and 3: Figures S2a, b and S3a, b, respectively.

### Evaluation of GLU and CK/GLU ratio in patients with early and metastatic BC

**Fig. 3:**
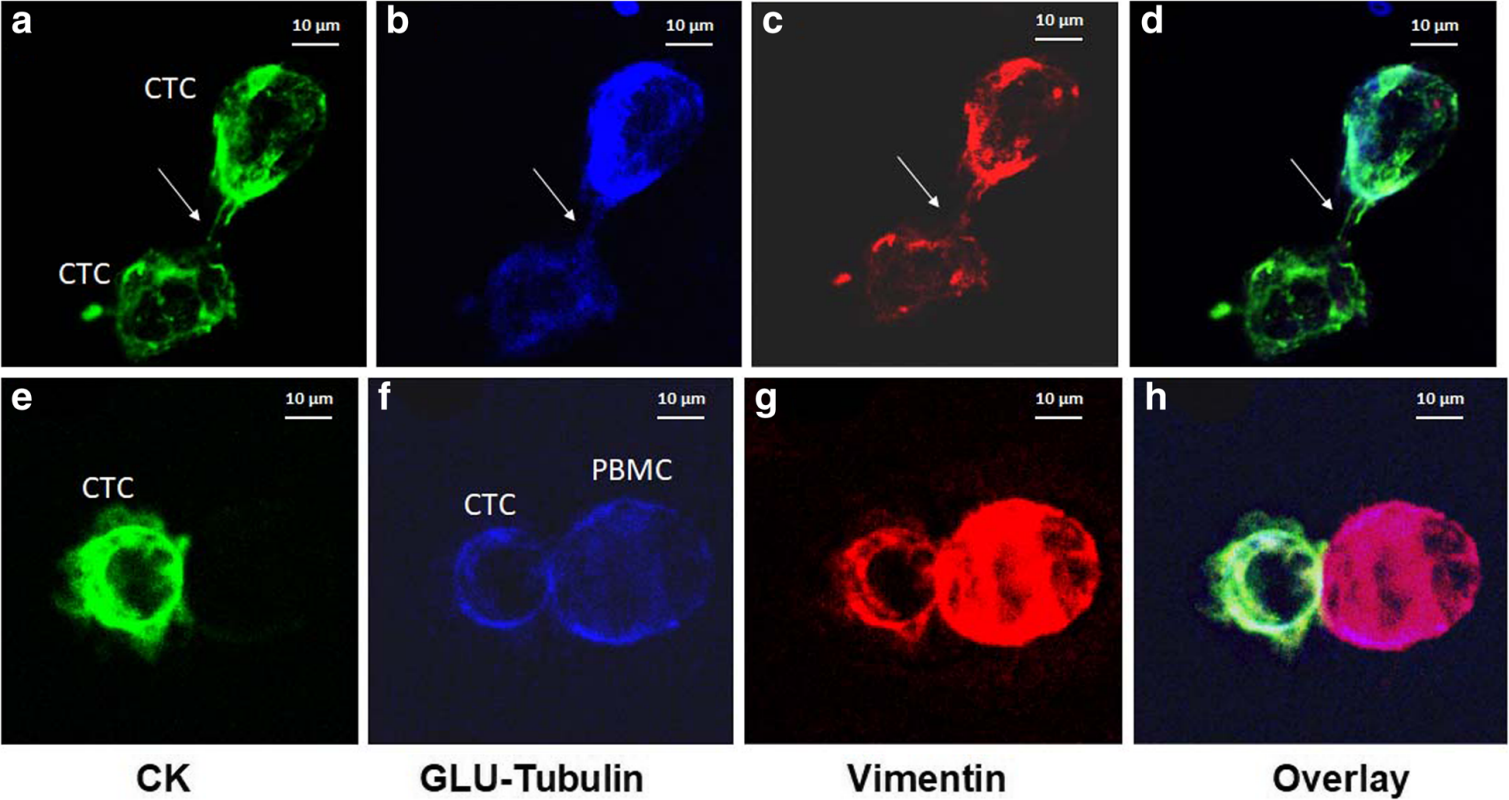
Expression of cytokeratin (CK), vimentin (VIM), and detyrosinated α-tubulin (GLU) in patients’ circulating tumor cells (CTCs). Patients’ samples were stained with pancytokeratin (A45-B/B3, green), detyrosinated tubulin (blue), and vimentin antibodies (red) and 4′,6-diamidino-2-phenylindole (DAPI, not shown). **a**–**d** Representative confocal laser scanning micrographs of patients’ CTCs (× 60) stained with pancytokeratin (A45-B/B3), vimentin, and GLU antibodies. Intercellular connections through cytoskeletal bridges (*white arrows*) were observed between CTCs. These microtentacles were supported by GLU, VIM, and CK. **e**–**h** CK, VIM, and GLU expression on a patient’s CTC (× 60), which is in contact with a peripheral blood mononuclear cell from a patient sample

Our results revealed that GLU participates in intercellular connections among patients’ CTCs (Fig. 3). GLU was expressed in a high percentages of patients with early and metastatic BC. The GLU^+^VIM^+^CK^+^ phenotype could be identified in 54.5% (6 of 11) of patients with early BC and in 62.5% of patients with metastatic disease (10 of 16) (Fig. 2a). In addition, GLU^+^VIM^−^CK^+^) CTCs could be detected in both patients with early BC (2 of 11 patients; 18.2%) and patients with metastatic disease (6 of 16 patients; 37.5%) (*p =* 0.69). Conversely, the phenotypes without GLU expression prevailed in an adjuvant setting compared with metastatic BC (GLU^−^VIM^+^CK^+^) (27.3% [3 of 11] vs 13% [2 of 16; *p =* 0.357). In addition, the GLU^−^VIM^−^CK^+^ phenotype was detected in 36.36% (4 of 11) of patients with early BC and in 13.33% (2 of 16) with metastatic disease (*p =* 0.357) (Fig. 2a).

The ratio CK/GLU was significantly higher in MCF7 cells (20.41 ± 0.6; *p =* 0.0001) than in CTCs from patients with metastatic BC (15.28 ± 2.8) (Table 2). Median intensity of GLU per patient was significantly increased in patients with metastatic BC compared with patients with early disease (5.29 ± 0.6 in early vs 6.99 ± 0.4 in metastatic setting; *p =* 0.029) (Fig. 2b); however, the ratio of CK/GLU (Fig. 2c) did not reach statistical significance (15.5 ± 0.6 vs 15.28 ± 2.8; *p =* 0.937). The distribution of all the CTCs regarding GLU intensity and CK/GLU ratio is shown in Additional files 2 and 3: Figures S2c, d and S3c, d.

### Evaluation of VIM and CK/VIM ratio in patients with early and metastatic BC

The intensity of VIM in CTCs detected in patients with metastatic BC (8.24 ± 1) was statistically higher than in MCF7 (4.27 ± 0.5; *p =* 0.0001) and SKBR3 (4.94 ± 0.4; *p =* 0.0001) cells (Table 2). In addition, the intensity of VIM in patients with early (5.38 ± 0.3; *p =* 0.022) and metastatic disease (8.24 ± 1; *p =* 0.0001) was also significantly higher than in MCF7 cells (Table 2). Mann-Whitney analysis also revealed significantly increased VIM expression in CTCs from patients with metastatic BC (8.24 ± 1) compared with early disease (5.38 ± 0.3) (*p =* 0.0001) (Fig. 2b).

CK/VIM ratio was lower in CTCs, regardless of disease stage, than in MCF7 cells (Table 2). In addition, the CK/VIM ratio was significantly lower (Fig. 2c) in patients with metastatic BC (8.05 ± 1.9) than in those with early disease (14.33 ± 0.6, *p =* 0.007).

There was also a positive correlation between the CK/TUB and CK/GLU ratios in CTCs (Spearman’s correlation analysis; *p =* 0.011). In addition, there was a positive correlation between CTC phenotypes TUB^+^VIM^+^CK^+^ and GLU^+^VIM^+^CK^+^ (*p =* 0.001) in patients with metastatic BC. Moreover, as shown in Table 3, only one patient with metastatic BC and one in the early BC group harvested both GLU^+^VIM^+^CK^+^ and GLU^−^VIM^+^CK^+^ CTCs in their blood. Conversely, in the rest of the patients, all the CTCs were either positive or negative for these phenotypes. The distribution of all the CTCs regarding VIM intensity and CK/VIM ratio identified in both settings is shown in Additional file 2: Figure S2e and f and Figure S4e and f).

### Evaluation of TUB, GLU, and VIM in sequential samples from a patient with BC

**Fig. 4:**
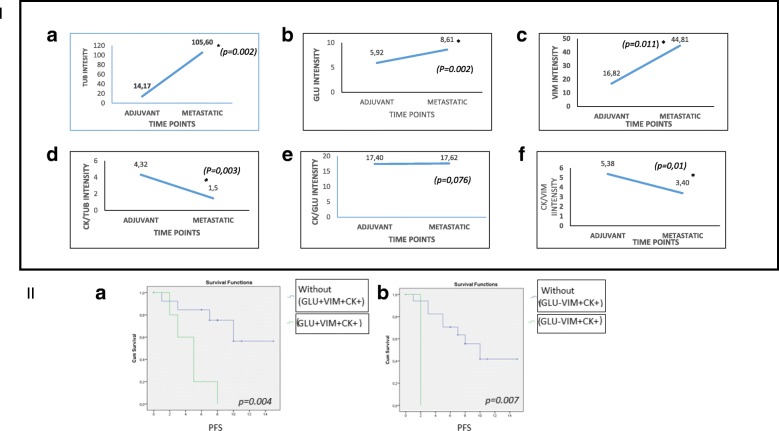
Progression-free survival (PFS) of patients with metastatic breast cancer. (**I**) Sequential samples from a patient with breast cancer. Quantification of α-tubulin (TUB) intensity (**a**), detyrosinated α-tubulin (GLU) intensity (**b**), vimentin (VIM) intensity (**c**), cytokeratin (CK)/TUB ratio (**d**), CK/GLU ratio (**e**), and CK/VIM ratio (**f**). (**II**) (**a**) PFS in patients with CK^+^GLU^+^VIM^+^-expressing CTCs (*p =* 0.004). (**b**) PFS in patients with CK^+^GLU^−^VIM^+^-expressing CTCs (*p =* 0.007)

During this study, one patient with early BC relapsed and developed metastatic disease. Therefore, it was possible to analyze two different blood draws during the course of the disease. The first blood sample was obtained before any clinical or imaging evidence of relapse, whereas the second corresponded to the time of documentation of metastatic disease. In accordance with our previous observations, TUB’s intensity in CTCs was statistically increased in the metastatic sample (*p =* 0.002) (Fig. 4Ia). In addition, the CK/TUB ratio was progressively statistically reduced (*p =* 0.003) (Fig. 4Id). Similarly, the intensity of GLU expression in CTCs was statistically increased (*p =* 0.002) between baseline and the time of relapse (Fig. 4Ib). The ratio CK/GLU was not significantly altered (*p =* 0.076) (Fig. 4Ie). Finally, there was also a statistical increase in VIM expression in CTCs (*p =* 0.011) when the patient’s BC became metastatic (Fig. 4Ic). The CK/VIM ratio was also statistically decreased (*p =* 0.01), between the first and the second sample draws (Fig. 4If).

### CTC phenotypic profile and clinical outcome

Although this study was a small pilot study and the results regarding clinical outcome are only exploratory, we analyzed the available clinical data from 22 of 24 patients with metastatic BC enrolled in this study. After a median follow-up of 8 months (range, 0–21), six patients (25%) had died. Three of them presented with overt metastasis at the time of initial diagnosis, whereas the rest presented with early BC. Patients who died during the follow-up harbored more CTCs (mean, 15; median, 9.5; range, 0–54) than survivors (mean, 2.31; median, 1; range, 0–15); however, survival analysis for total CTCs per patient (Cox regression *p* = 0.142, Kaplan-Meier *p* = 0.124) did not show statistical differences in patient outcomes. On the other hand, survival analysis regarding distinct phenotypes revealed that median PFS was 3.0 months for the patients with detectable GLU^+^VIM^+^CK^+^-expressing CTCs compared with 7.5 months for patients who did not have detectable CTCs with this phenotype (*p =* 0.004) (Fig. 4IIa). Similarly, patients with detectable GLU^−^VIM^+^CK^+^-expressing CTCs had a median PFS of 1.0 month compared with 7.0 months for patients who did not have detectable CTCs with this phenotype (*p =* 0.007) (Fig. 4IIb).

Finally, the ORR was significantly lower in patients with TUB^+^VIM^+^CK^+^-expressing CTCs compared with patients without CTCs bearing this phenotype (*p =* 0.046). Moreover, the ORR was significantly lower in patients with metastatic disease who had CTCs with high numbers of microtentacles (5 of 16 patients with CK^+^) (*p =* 0.019). The criteria for the evaluation of ORR were according to RECIST 1.1: tumor size, lymph node status, lesion number, and so forth [32].

## Discussion

It is widely accepted that although CTCs hold a crucial role in the metastatic process, the changes occurring during disease evolution on these cells are not fully characterized yet. CTCs hold significant prognostic value for patients with both early and metastatic BC [33–35]. It is also well known that microtentacles are increased in aggressive BC cells compared with less invasive phenotypes [19]. Microtentacles are supported by TUB, GLU, and VIM [19, 20]. In the current study, we investigated the presence of those filamentous protrusions in CTCs isolated from early and metastatic patients with BC. Moreover, the expression of the implicated molecules was quantified, and their association with the patients’ clinical outcomes was assessed.

Our experiments revealed that microtentacles supported by TUB could be detected in patients early BC but mainly in those with metastatic BC. Interestingly, patients with increased numbers of microtentacle-presenting CTCs experienced a significantly lower ORR than patients with a low number of microtentacles (*p =* 0.019), suggesting that their tumors were more resistant to treatment. Furthermore, the results of the current study demonstrated that CTCs could contact each other (Figs. 1 and 3, white arrows) with filamentous protrusions supported by TUB, VIM, and GLU. These protrusions were also observed to connect CTCs with blood cells; however, in such junctions, only TUB and VIM were present, whereas CK expression was limited (Figs. 1 and 3). These findings are in agreement with previous observations in two other studies in which researchers reported that CTCs could be accompanied in the vessels by blood cells (giant macrophages) that seem to be associated with an unfavorable clinical outcome [36, 37]. Therefore, our results could potentially explain how CTCs may travel in the bloodstream next to blood cells.

In our quantification of the ratios CK/TUB, CK/GLU, and CK/VIM in three representative subtypes of BC (MCF7 [HR^+^], SKBR3 [HER2^+^], and MDA-MB-231 [basal-like]), we observed that these ratios were statistically increased in MCF7 compared with SKBR3 and MDA-MB-231 cells. This suggests that they can be used as distinct markers to characterize less aggressive tumor cells, such as the HR^+^ subtype from more invasive phenotypes. In addition, microtentacles were observed mostly in MDA-MB-231 cells (Additional file 1: Figure S1). These findings support previous studies in that basal-like cell lines have increased numbers of microtentacles [38]. Furthermore, MDA-MB-231 cells have significantly higher levels of VIM intensity (28.87 ± 3.7) than other cell lines (Additional file 2: Figure S2, Table 2), in accordance with previously published data [39].

Characterization of CTCs on the basis of these markers clearly indicated that CK/TUB and TUB intensity were statistically different in early BC compared with the metastatic setting (Table 2), implying that during disease evolution, the level of TUB increases relative to cytokeratin intensity. This observation could be a useful tool for the characterization of CTCs with more aggressive features, mostly in patients with early BC, where the cells are very heterogeneous [12]. The analysis of sequential samples from a patient with BC during the early and metastatic stages of the disease confirmed the previous observations. Indeed, during the course of the disease, the expression of TUB was significantly increased (Fig. 4a), whereas the CK/TUB ratio was decreased (Fig. 4d). These results strongly suggest that follow-up samples regarding TUB expression in CTCs could potentially give useful information about disease relapse before the appearance of clinical and laboratory findings of overt metastasis.

VIM intensity was also statistically increased in the basal-like cell line MDA-MB-231 compared with HR^+^ MCF7 cells (Table 2). Consequently, the ratio of CK/VIM was also decreased in the aggressive cell line compared with HR^+^ cells (Table 2). These results imply that VIM is also characteristic of more aggressive phenotypes, in accordance with previous studies [39]. These observations were confirmed in patients’ samples, indicating that VIM intensity was statistically increased in metastatic (*p* = 0.0001) compared with early disease, whereas the CK/VIM ratio was significantly decreased (*p* = 0.007) in patients with advanced BC (Table 2).

Furthermore, although the CK/GLU ratio did not reach statistical significance between early and metastatic disease (Table 2), the intensity of GLU was significantly higher (*p =* 0.029) in patients with metastatic BC than in those with early BC (Table 2). These findings strongly suggest that GLU can also be a marker characterizing invasive subpopulations. In accordance with this, the presence of the CK^+^GLU^+^VIM^+^ phenotype in patients’ blood was associated with worse PFS (*p =* 0.004), suggesting that both markers (GLU and VIM) could represent poor prognosis factors when coexpressed in patients’ CTCs. Interestingly, the total number of CTCs did not correlate with prognosis in patients with metastatic BC, implying that the characterization of distinct phenotypes is critical for disease outcome, as we have previously shown [16]. This assumption is in line with previous studies regarding GLU expression in primary tumors [25]. However, this is a pilot study with a small number of patients; therefore, our results are only exploratory. A larger study with an increased number of patients is needed to confirm our observations.

It was also interesting that Spearman’s correlation analysis revealed a correlation between CK/TUB and CK/GLU ratios (*p =* 0.005) in CTCs. In addition, CK/GLU ratio was also significantly correlated to CK/VIM ratio (*p =* 0.011), implying that all these markers can be used concomitantly to underline an aggressive signature of CTCs in patients with BC. Finally, it is noteworthy that all these molecules were present in the junctions among CTCs, mostly in patients with CTC clusters, and it is of interest that these patients have a 50-fold increased risk of relapse [40].

## Conclusions

Microtentacles observed in CTCs isolated from patients with BC participate in the communication among CTCs and the interaction between CTCs and blood cells. The proteins that support these protrusions (TUB, VIM, and GLU) potentially represent markers for the identification of CTCs with a more aggressive phenotype in patients with BC; however, a study with a larger group of patients is necessary to further confirm the clinical relevance of these findings.

## Additional files

Additional file 1: **Figure S1.** Expression of cytokeratin, vimentin, and α-tubulin on MCF7, SKBR3, and MDA-MB 231 cells spiked in normal blood and isolated with the ISET system. Representative confocal laser scanning micrographs of MCF7 (× 60), SKBR3 (× 40), and MDA-MB 231 (× 40) cells, triple-stained with pancytokeratin (A45-B/B3), vimentin, and α-tubulin antibodies. (JPG 117 kb)

Additional file 2: **Figure S2.** Single CTC distribution regarding TUB, VIM, and GLU intensity. TUB expression in CTCs obtained from patients with (**a**) early and (**b**) metastatic breast cancer. Each dot represents the intensity of one CTC. GLU expression in CTCs obtained from patients with (**c**) early and (**d**) metastatic breast cancer. Each dot represents the intensity of one CTC. VIM expression in CTCs obtained from patients with (**e**) early and (**f**) metastatic breast cancer. Each dot represents the intensity of one CTC. (JPG 122 kb)

Additional file 3: Single CTC distribution regarding CK/TUB, CK/VIM, and CK/GLU ratios. CK/TUB ratio in CTCs obtained from patients with (**a**) early and (**b**) metastatic breast cancer. Each dot represents the intensity of one CTC. CK/GLU ratio in CTCs obtained from patients with (**c**) early and (**d**) metastatic breast cancer. Each dot represents the intensity of one CTC. CK/VIM ratio in CTCs obtained from patients with (**e**) early and (**f**) metastatic breast cancer. Each dot represents the intensity of one CTC. (JPG 130 kb)

## Abbreviations

CTC: Circulating tumor cell
EDTA: Ethylenediaminetetraacetic acid
EMT: Epithelial-to-mesenchymal transition
ER: Estrogen receptor
GLU: Detyrosinated α-tubulin
HER: Human epidermal growth factor receptor 2
HR: Hormone receptor
IgG1: Immunoglobulin G1
ORR: Objective response rate
OS: Overall survival
PFS: Progression-free survival
PR: Progesterone receptor
RT: Room temperature
TUB: α-Tubulin
VIM: Vimentin
