# Supplementary figures and images for: Evaluation of α-tubulin, detyrosinated α-tubulin, and vimentin in CTCs: identification of the interaction between CTCs and blood cells through cytoskeletal elements
Source: Breast Cancer Res. 2018 Jul 5;20:67. doi: 10.1186/s13058-018-0993-z (PMC6034292; doi:10.1186/s13058-018-0993-z)

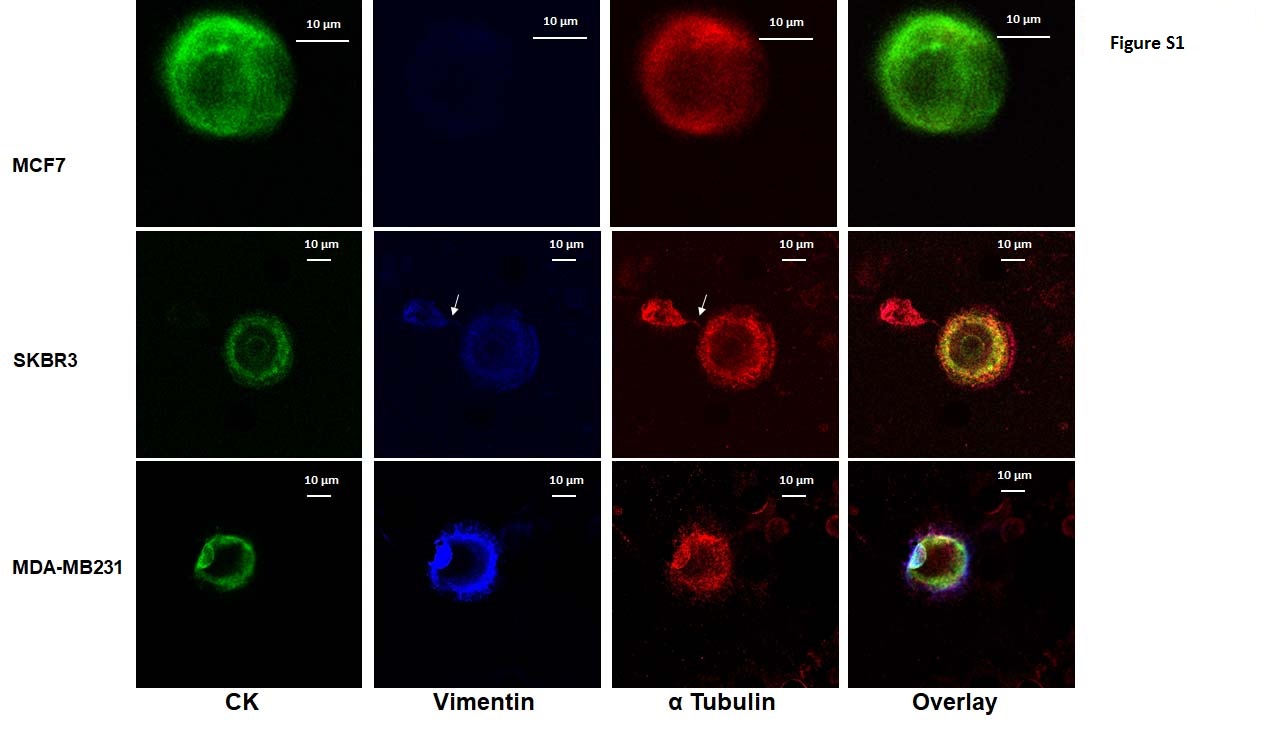

Supplement: Supplementary file 1 — Figure S1. Expression of cytokeratin, vimentin, and α-tubulin on MCF7, SKBR3, and MDA-MB 231 cells spiked in normal blood and isolated with the ISET system. Representative confocal laser scanning micrographs of MCF7 (× 60), SKBR3 (× 40), and MDA-MB 231 (× 40) cells, triple-stained with pancytokeratin (A45-B/B3), vimentin, and α-tubulin antibodies. (JPG 117 kb) [file 13058_2018_993_MOESM1_ESM.jpg]

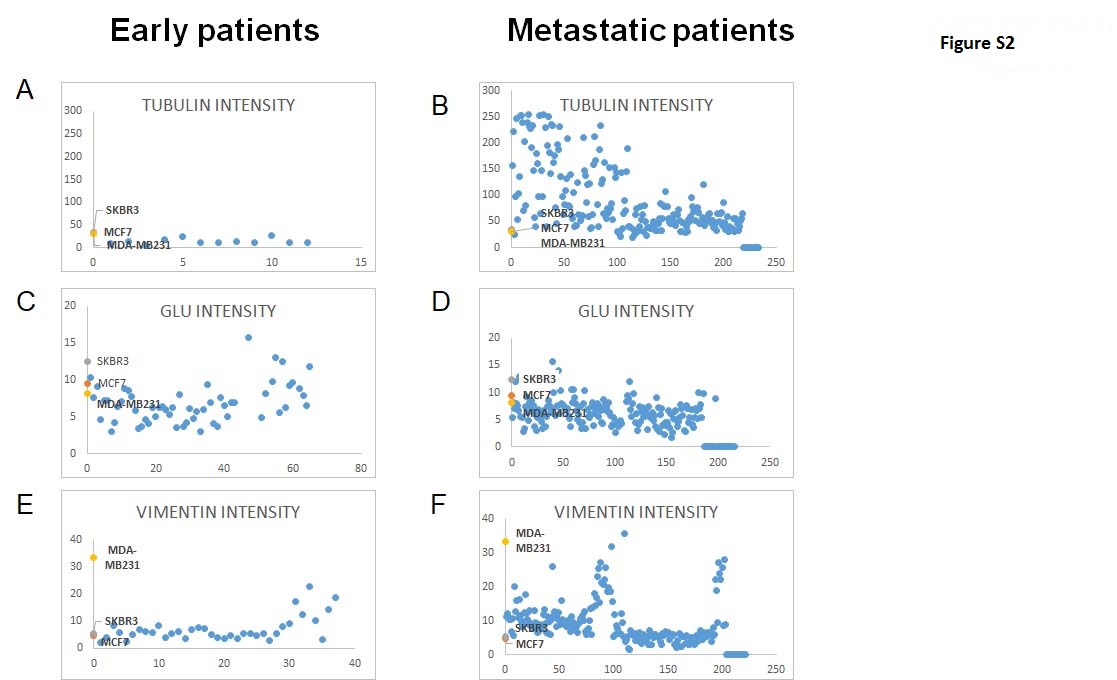

Supplement: Supplementary file 2 — Figure S2. Single CTC distribution regarding TUB, VIM, and GLU intensity. TUB expression in CTCs obtained from patients with (a) early and (b) metastatic breast cancer. Each dot represents the intensity of one CTC. GLU expression in CTCs obtained from patients with (c) early and (d) metastatic breast cancer. Each dot represents the intensity of one CTC. VIM expression in CTCs obtained from patients with (e) early and (f) metastatic breast cancer. Each dot represents the intensity of one CTC. (JPG 122 kb) [file 13058_2018_993_MOESM2_ESM.jpg]

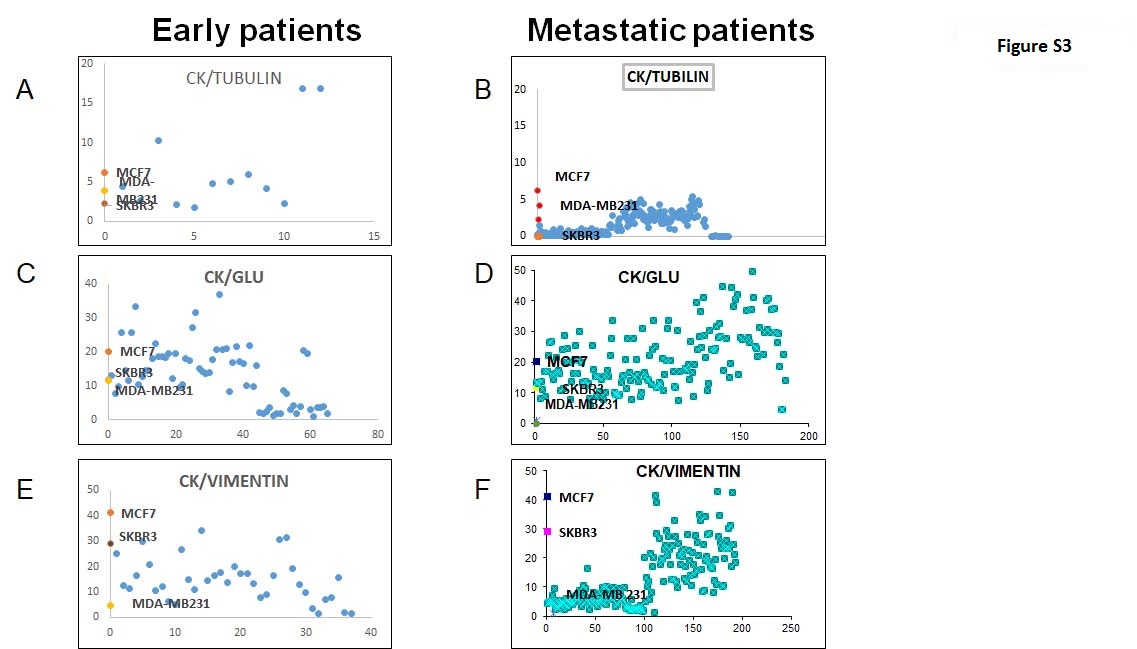

Supplement: Supplementary file 3 — Single CTC distribution regarding CK/TUB, CK/VIM, and CK/GLU ratios. CK/TUB ratio in CTCs obtained from patients with (a) early and (b) metastatic breast cancer. Each dot represents the intensity of one CTC. CK/GLU ratio in CTCs obtained from patients with (c) early and (d) metastatic breast cancer. Each dot represents the intensity of one CTC. CK/VIM ratio in CTCs obtained from patients with (e) early and (f) metastatic breast cancer. Each dot represents the intensity of one CTC. (JPG 130 kb) [file 13058_2018_993_MOESM3_ESM.jpg]
